# Listening to stakeholders in the prevention of gender-based violence among young people in Spain: a qualitative study from the positivMasc project

**DOI:** 10.1186/s12905-023-02545-3

**Published:** 2023-07-26

**Authors:** Jorge Marcos-Marcos, Krizia Nardini, Erica Briones-Vozmediano, Carmen Vives-Cases

**Affiliations:** 1 Department of Health Psychology, University of Alicante, Alicante, Spain; 2 Department of Community Nursing, Preventive Medicine and Public Health and History of Science, University of Alicante, Alicante, Spain; 3 Faculty of Nursing and Physiotherapy, University of Lleida, Lleida, Spain; 4 Center for Biomedical Research in Epidemiology and Public Health (CIBERESP), Madrid, Spain

**Keywords:** Gender-based violence, Primary prevention, Masculinities, Stakeholders, Qualitative research, Spain

## Abstract

**Objective:**

This study seeks to deepen current knowledge of the phenomenon of gender-based violence (GVB) among young people in Spain, identifying the main challenges in terms of prevention from the perspective of key stakeholders in the field.

**Methods:**

23 semi-structured qualitative interviews were performed with professionals whose work involves youth and comes from different areas: social work, policy making, youth education, feminist and LGBTQ activism and anti-violence masculinities engagement (13 women and 10 men).

**Results:**

Among the main challenges identified by stakeholders in relation to GBV preventive strategies in young populations there is a need to focus on transformative programmes within educational settings. The findings indicate that specific programs and interventions in this area may not be yielding the expected effectiveness. This outcome could be attributed less to a lack of resources and more to a failure to address the core issues and challenges adequately. Thus, the results underline that intervention programmes should emphasise equitable gender norms and gender relations and incorporate content on anti-violence masculinities. Finally, a pivotal aspect seen by professionals to facilitate GBV prevention is the design and development of interventions based on participatory and active approaches, close to young people’s everyday situations. The results also draw attention to the need to analyse the impact of new forms of violence in greater depth, especially those that occur through information and communication technologies.

**Conclusion:**

Among other implications for policy and practice, the study points to the need to articulate interventions designed to work simultaneously at different levels of influence acting on people.

**Supplementary Information:**

The online version contains supplementary material available at 10.1186/s12905-023-02545-3.

## Background

Gender is a widely accepted social determinant of health. The inclusion of gender equality as a goal in the United Nations Sustainable Development Goals has reinforced the promotion of equitable relationships, but also the preventive implications in relation to gender-based violence [1]. Although theories and programmes for primary prevention are relatively recent in the field of gender-based violence (GBV), a growing body of research shows that gender-focused interventions can lead to reductions in violence and other positive health outcomes [2].

When operating within a specific context, literature shows that implementing interventions simultaneously across multiple levels (ranging from individual to societal) is more effective than solely focusing on interventions at a single level [3, 4]. In the case of young populations, it becomes particularly vital to work cohesively at both the individual and interpersonal levels, considering the influential role of stereotypes and social norms in shaping behaviors, including patterns of gender-based violence [5]. Likewise, this emphasizes the significance of examining gender identities and relationships in relation to the intricate nature of their perceptions, attitudes, and beliefs surrounding GBV [6–8].

In terms of understanding violence itself, literature shows that young people define violence as encompassing a wide range of behaviours, from physical acts to emotional abuse and control [9]. This has focused mainly on frameworks based on established behaviour change theories, especially in relation to the psychosocial determinants of behaviour, positing how environmental and programme inputs lead to output behaviours via structural, interpersonal and individual processes [10].

The relevance of challenging harmful power imbalances as a catalyst for change in relation to GBV has put the spotlight on gender-transformative approaches [11]. These programmes, focusing on the critical evaluation of social norms and gendered expectations, as well as on promoting gender-equitable behaviours and attitudes [12], pay special attention to the need to address masculinities, that is, how “normative” gendered traits affect attitudes in men’s lives, but understanding them primarily as configurations of collective practices, thus avoiding limiting gender analysis to a review of individual personality traits [13–15]. In fact, programmes have usually pivoted on this issue since the use of violence by men and boys has been consistently associated with adherence to hegemonic notions of masculinity that emphasises control over and hostility towards women and girls [16, 17]. Nevertheless, while it has been emphasized that men’s gender identity should not be treated as a homogenous group, neglecting the heterogeneity of their life experiences, extensive scientific literature has consistently demonstrated that social expectations associated with conforming to traditional notions of masculinity often involve adopting harmful behaviors characterized by resistance, aggression, and engagement in risky behaviors [18, 19]. Additionally, there is often a lack of “emotional literacy,“ manifesting as limited understanding, skills, and knowledge regarding one’s own emotions and those of others. This deficiency has been linked to challenges in effectively managing conflicts, establishing healthy relationships, making informed decisions, and coping with stress [20, 21], which are understood as a problem for both women and men in terms of well-being and are a fundamental cause of inequalities in health [22]. Literature shows that gender-transformative approaches provide promising outcomes in preventing GBV perpetration and health risk behaviours, shifting to a greater extent men’s gender and violence-related behaviours and attitudes than other programmes that do not address concepts about gendered social norms [11, 12, 23]. In this regard, a recent systematic review has also revealed that this gendered approach could be promising in educational interventions that involve men and boys to reduce GBV by promoting positive masculinities [24].

We are facing a key moment in the process of building knowledge and critical thinking about the impact of GBV. In this process, it is crucial to consider the sociocultural contextualization of prevention efforts and favouring approaches that take into account the gender roles and expectations in which people’s real lives unfold.

This study, carried out in Spain, is based on the premise that achieving changes requires focusing not only on attitudes, behaviours and/or gendered stereotypes at an individual and interpersonal level, but also on a person’s surroundings (e.g. school, family, community, social media, etc.), and to enhance the transfer of research to practice, it is necessary to strengthen the engagement of key stakeholders [4, 25]. This idea took on particular significance within the context of the study following the legislative and social advancements in addressing gender violence, culminating in the approval of Spain’s Organic Law 1/2004 on Integral Protection Against Gender Violence. This law recognizes gender violence as a form of discrimination and a consequence of unequal power dynamics between women and men who are (or have been) intimate partners or have had emotional connections, even without cohabitation [26, 27]. As a result, various initiatives and institutions have been established, including the Observatory, the implementation of the VioGén system, specialized Courts for Violence against Women, and the creation of ATENPRO, a dedicated victim protection service. Furthermore, the commitment to addressing this issue is evident through the legal frameworks and institutional responses in place, such as the ratification of the Istanbul Convention on August 1, 2014, and the formulation of several action plans and political initiatives. Notably, the current “State Strategy to Fight Against Sexist Violence 2022–2025” underscores the ongoing dedication to combatting gender-based violence. However, the statistics show a growing trend in recent years: the greatest increase in both the number of victims and the number of reported cases occurs among young people [28].

This study has been designed to deepen the current knowledge of the phenomenon of GVB among young people from the perspective of stakeholders, identifying the main challenges in terms of prevention. Thus, the article focuses on giving a voice to professionals working with young people in different fields. This is a collective that, in the context of the study, has so far received little attention in the development of public initiatives to reduce this type of violence from an early age. This article specifically aims to examine the prevailing discourses identified by stakeholders regarding barriers to raising awareness about gender-based violence (GBV) and the strategies for constructing a discourse on GBV prevention among young people. The potential significance of this paper lies in exploring stakeholders’ perspectives on areas where current GBV prevention measures may be ineffective and the underlying reasons for them.

## Methods

This qualitative study was conducted between October 2019 and February 2020 in Madrid (Spain) and is part of the PositivMasc EU research project [29]. The aim of PositivMasc is to explore the relations among youth, masculinities and violence against women (VAW) in order to inform on the development of strategies and resources to promote anti-VAW masculinities in Sweden, Ireland, Israel and Spain.

### Sample

The participants of this study were 23 stakeholders (13 women and 10 men) who work in the field of GBV prevention in Spain: social workers (4), policy makers (4), youth community educators (4), feminist and LGBTQ activists (4) and professionals working on masculinities (7). They were recruited purposely by directly contacting both governmental and non-governmental organisations involved in the prevention of GBV, with the aim of covering areas of intervention and decision-making on this matter among the young people. In the case of NGOs and other civil society actors, which play an important role in the scope of the study, their work was carried out mainly through grants and agreements with public institutions for the provision of services. In order to reach more contacts in the field, we also used the snowball technique where interviewees provided other significant contacts in the field. Together they represented a non-probability purposive sampling based on typical cases [30].

### Data collection

In total, we conducted 23 semi-structured interviews. The data gathering process ended by following the principle of saturation [31]: i.e., when discourses provided no additional information on the issue under study and when all the areas of work in GBV prevention and youth were covered.

The interviews were designed to gather discourses around stakeholders’ experiences and perspectives about their work on GBV-prevention among young people. The focus of the interviews was set on understanding current challenges in GBV prevention work, youth’s responses to such work and the relations between GBV and perceived gender norms (femininities and masculinities). Additionally, interviews lacked and improvements for Interviews’ content was developed based on a literature review and on preliminary discussions with national advisory groups of the PositivMasc project, including stakeholders working with youth in GBV prevention. Interviews addressed participants’ work with young people about: (a) new forms of GBV, (b) barriers and challenges in the field, (c) effective strategies and (d) urgent issues to address when working with young people and GBV prevention. In any case, the topic guide was applied with sufficient flexibility to allow new questions to emerge during each interview. The duration of each interview ranged from 45 to 90 min. All the interviews, previously conducted in Spanish, were analysed in the original language by the research team; then, selected quotations were translated into English by the team at the moment of writing the manuscript.

### Data analysis

All interviews were recorded and transcribed verbatim in its original language. We used discourse analysis [32, 33]: as the most adequate method to qualitatively explore participants’ experiences and standpoints. Discourse analysis allowed us to grasp different professionals’ perspectives in relation to their work on youth and GBV prevention. We understand discourse as a realm in which discursive practices cannot be disentangled from material and social practices [34, 35]. Thus, we consider that all the discursive elaborations in the data collection concern masculinities and femininities and discuss what men do “as men” and what women do “as women” in specific situations.

The discourse analysis was carried out by inductively coding the text of the transcriptions and constantly comparing data until we could group the codes into families according to their similar meanings. This constant comparative approach allowed us to identify the patterns across discourses and what axes of variation were present in the data. The coding process was conducted and reviewed by three members of the research team in order to increase the reliability of the analysis process. The trustworthiness of the information generated by the qualitative data and its relationship to the established categories and codes were evaluated in terms of saturation. Thus, only data that met this criterion were included in the results. The ATLAS.ti computer software was used both to set the emerging discourses and to facilitate the triangulation process between the different research team members independently. An additional file shows the coding tree in detail [see Additional file 1].

### Ethical issues

The research was carried out in accordance with the ethical principles of the Declaration of Helsinki [36]. Thus, all participants were informed about the research objectives, ethics and general framework of the project prior to participating. Participation was voluntary and all participants had the option to withdraw from the study at any time. Written informed consent from the study participants was obtained prior to enrolment. Confidentiality was guaranteed in adherence to the European Union General Data Protection Regulation (2016/679). The study was approved by the ethical committee of the University of Alicante (UA 2019-04-15).

## Results

Four key lines of discourse were identified based on two major analytical categories: (a) prevalent discourses related to barriers to GBV awareness in young people, which are linked to (mis)understanding gender relations and (un)recognized forms of intimate partner violence among young people and (b) strategies to construct a GBV prevention discourse that could offer prevention beyond raising awareness and with gender-transformative approaches.

### Barriers to gender-based violence awareness in young people

#### Gender-based violence and (mis)understanding gender relations

When discussing GBV, professionals encountered a general agreement among young people about the need to eradicate violence and support gender equal relations. However, even considering a significant level of awareness on GBV among the youth, professionals revealed some contradictions: they considered that some young people stand against GBV while not recognizing certain practices as affecting or being affected by GBV. In fact, the professionals stressed the idea that we are witnessing a generation of young people that generally shows discourse of conscience regarding the GBV prevention, but this discursive awareness is not guaranteeing whether they, both men and women, understand the mechanisms of action of the different forms of violence, and this is something the programs are not addressing. Among these forms of violence that are not understood, are those highlighted sexual harassment.

*There are many young girls that, on a level of experiencing violence against them, do not conceptualize it in all its… [forms]. You explain it to them and based on the discourse they tell you “because insistence is violence”. If we are talking about sexual violence, they do know that a sexist relationship is within the patriarchal logic of seduction, etc. Nonetheless, those who are not aware clearly say that it is normal to insist, it is something that is normal in nightlife, that is what it is like* (Interview 5, woman, expert in GBV and youth educator, representative at a women’s association).

Among the barriers identified by professionals to progress in this matter, they highlighted that the social construction of gender relations is not clearly integrated in the interventions and programmes that are developed. This has facilitated, according to the interviewees’ perspectives, that young people’s awareness about GBV prevention and GBV rejection coexist, for instance, with the lack of recognition about how sexism and gender norms contribute to GBV, which is another content that the professionals considered must be strengthened within the actions developed. In the same way, the professionals also underlined that programmes and interventions should be designed bearing in mind that we are facing a complex social phenomenon crossed by significant gender inequalities. While the professionals interviewed acknowledged the presence of some young individuals with a strong anti-violence stance and an open-minded perspective, they regarded these youths as exceptions. Notably, the professionals observed that girls exhibit a clearer awareness than boys regarding the influence of sexist norms on both their own upbringing and that of boys. This observation led the professionals to conclude that, overall, men are “lagging behind” in recognizing and challenging harmful gender norms, although they acknowledged the existence of exceptions, including men and boys who may also be victims of perpetrators. The following interview excerpt alludes to how this is also related by professionals to the fact that many young men still perceive gender as something alien to them.

*They do not know how to act in a gender violence situation, because they do not consider it beyond what they are told: “you do not act, because something can go wrong”. They do not see alternatives; they do not understand that feminism is not just a matter of women.* (Interview 14, man, expert in GBV and youth educator, representative at a non-governmental association)

When contemplating the gendered disparities and the obstacles that hinder swift progress in understanding gender-based violence (GBV), the professionals noted that this might be connected to the acceptance and perceived benefits, from the perspective of men and boys, of remaining silent on GBV and refraining from intervening in such matters. This dynamic exists alongside the potential repercussions faced by those who are willing to take action, knowing that they themselves may become victims. Nevertheless, within this complex scenario, the professionals highlighted another crucial aspect that programs and interventions should explicitly address: how maintaining privileges could be playing a capital role in this issue, avoiding feeling responsible for inequalities related to GBV.

*We, boys, continue to associate gender violence with aggression, with hitting, with direct violence. The invisible variable of this violence is not considered, which is cultural and structural (…) identifying different forms of machismo is perceived more in girls than in boys. The problem is there is a rejection because as the final fact of sexual violence is what stands out, which is rape as a physical act, well I do not identify with it.* (Interview 19, man, expert on masculinities and gender equality, representative at a non-governmental association).

*We are not that stupid, we recognize it as violence, but we win if it is not recognized as violence, as in the discourse it is not [included] unless extreme cases are reached. Even in extreme cases we are assumed to be innocent and in that discourse “she has asked for it”, “what did she do”. We live in a very comfortable society where it is never our fault. When you stop to analyse it, you think “God, in reality I am creating all this circle of violence”, right?* (Interview 15, man, expert on equality and masculinities, representative at a non-governmental association).

To advance the promotion of gender privilege awareness, professionals emphasized the importance of not only incorporating these topics into programs but also reevaluating approaches to unveiling the unseen. For instance, certain professionals, particularly those affiliated with feminist organizations, specifically highlighted the advantages of having both men and women trainers leading the training. Two participants articulated this perspective, noting that hearing a man address gender-based violence tends to have a positive impact on both men and women.

*We wanted people to see that gender violence is an issue that affects us all. We wanted them not only to see women talking about it, but also to see a man talking about it, criticizing himself, so to speak, pointing out the mistakes we can all make, and I pointing out our own mistakes and he pointing out the mistakes of the male perspective. Precisely, in the evaluations we did at the end of each workshop, we saw that this was identified as something very positive by the men (…) in addition to the men seeing an alternative model, I think it can also be useful for the girls. Because seeing a man talk about these issues gives you a little bit of… how can I say? Even hope, that it is not something that only affects you, only we talk about it. But there are also men who are concerned about these issues* (Interview 10, woman, representative at a non-governmental association).

*We believe it’s crucial to not only share our perspective as women on what violence means to us or what abusive relationships entail but also to have a man speak to them about it. We’d love to have men participate in all the workshops, but it’s not always feasible (Interview 2, woman, representative at a non-governmental association).*

#### The (un)recognized forms of GBV among young people

The professionals observed violent attitudes and GBV among young people in forms that are not seen nor recognized as such. The discourse and imaginary of GBV shared among young people, according to the professionals’ opinion, is clearly influenced by the mass media, that tend to focus on physical violence. They considered the need to fight against these stereotypical narratives on GBV, as they do not facilitate young people’s self-reflection processes on how violence is part of their experiences and how it is reproduced. In fact, professionals indicated that although most girls are more aware than boys of the inner GBV dynamics involved in toxic practices in sexual-romantic relationships, a general acceptance of control practices in these relationships is found.

*It depends, there are many boys and girls that have this radar of “this is not right”, “this can be controlling”. However, many others do not. They believe jealously as something that is admissible, even something good.* (Interview 13, woman, expert on violence and masculinities, representative at a non-governmental association).

*The girls’ discourses are… “he says that if I do not do it, it is because I do not love him”, But, yeah, the difficult thing is for them to identify that as intimidation.* (Interview 8, woman, expert in GBV prevention, representative at a non-governmental association).

Although psychological violence is identified by young girls and boys, according to professionals the dominant imaginary equates GBV with physical violence and sexual abuse from a stranger. The following statements reveal that interventions must also address contents that are rarely considered forms of GBV: sexual abuse by partners and control exercised through digital technologies.

*I do see that we are progressing. We should of course be quicker, but psychological violence is now identified. But, of course, what is not identified as violence is structural or control behaviours, that is not identified. They still confuse it with romantic love.* (Interview 9, woman, GBV prevention worker).

*There is a bit of distortion between what they understand, or what the law understands, what is gender violence with a partner or ex-partner, jealousy, control, now we have realised that we also need to include lots of technology tools that are making these kind of aggressions change. Now there is another kind of violence in virtual spaces.* (Interview 5, woman, expert in GBV and youth educator, representative at a women’s association).

The professionals showed great concern regarding the new forms of GBV that occurred in digital communications, which is considered to be very common among young people and is being addressed in a limited way. This type of violence is especially visible within sexual-affective relationships and dating settings, when one partner exercises control over the other using digital technologies. According to the interviewees, these forms of control include asking for partners to share their geo-localization when they are not together, asking for passwords as a sign of romantic love or demanding access to each other’s communication devices and online accounts as a proof of trust. The following excerpts show that the professionals considered that cultural sexist myths of romantic love are being reinforced through these practices of control and jealousy, performed through and in relation to the use of digital communication platforms:

*Now we have seen that relationships have a very strong point of toxicity. Because when we talk about jealousy, there are always looks, nudges, taps under the table. With WhatsApp and controlling the phone.* (Interview 7, woman, GBV expert).

*Giving out passwords is normal. And then, there is also pressure and extortion with photos, the case is more men with women than the other way around. That is regarding the attitude towards social networks, regarding relations. And then if you ask them, they know what is politically correct.* (Interview 8, woman, GBV prevention worker).

When it comes to shedding light on these overlooked forms of violence and fostering the process of reflection, professionals mentioned the use of playful and interactive approaches in recreational spaces and activities as a pedagogical and methodological strategy. In the following excerpt, one of the professionals specifically highlights forum theater as a dynamic with significant potential for this type of intervention.

*We also do this thing called forum theater. It’s a pretty new format where there’s a play, usually about a violent relationship, with a bunch of young actors and actresses. We usually invite high school students to watch the play. But here’s the cool part: at certain points in the performance, we stop the play and invite the audience to come up and change the story a bit. It’s actually quite cathartic, you know, and some really awesome situations come up during that.* (Interview 23, woman, youth educator and expert in GBV).

### Enablers to construct a better GBV prevention discourse

#### GBV prevention strategies beyond raising awareness

The interviewed professionals agreed that it is necessary to prioritize the approach to gender norms as part of GBV prevention. In this regard, they considered it crucial to stress and unmask the social constructions related to sexual health, romantic relationships and dating culture but also the gendered division of labour. As stated in the following excerpts, stakeholders find it key to incorporate content on deconstructing gender expectations to raise awareness of the harmful beliefs and practices that underpin GBV, as well as to educate about the history of gender inequalities, including the role of the social production of hegemonic masculinities in all of this.

*When we work on gender construction, it is important to understand how the way of relating it has been constructed. And that can be based on hegemony, it is dependency. All of this works if everyone plays their role. (Interview 20, woman, expert on equality and masculinities, representative at a non-governmental association).*

According to the professionals’ perspective, interventions should focus primarily on understanding gender socialisation and its relation to violence, paying specific attention to working with men and boys to challenge the cultural roots that might underpin such violence.

*I focus on socialisation a lot (…) on how men’s subjectivity is constructed, women’s perception, self-perception, what it means to be a man. I would focus on that. All this experience of privileges implies, in the end, a socialization that is related to learning things: violence and values.* (Interview 18, man, equality and masculinities worker).

*The reason why it is important to work with men is simple and basic: they exercise violence. So, the role of men is crucial if we really want to break it.* (Interview 9,

woman, GBV prevention worker).

woman, GBV prevention worker).

The findings indicate that alongside the development of programs and interventions aimed at reshaping gender norms and enhancing individual and interpersonal skills, there is a need for a structural prevention approach that brings visibility and fosters critical awareness. The results underscore the significance of incorporating experiential activities that resonate with young people in terms of content and language. For instance, one participant highlighted the effectiveness of various actions and communication strategies implemented in public spaces with the support of local authorities.

*How do we get people to really grasp what we mean when we talk about male violence? One time, we tried something cool: we painted messages on crosswalks to spread awareness. And guess what? It worked! Now we’re planning to organize a graffiti course (…) The idea is to help people understand the whole deal with male violence. We’ll cover everything from micro-machismo to compliments that cross the line, basically anything that falls under violence against women (Interview 21, man, expert on masculinities and GBV).*

In order to make progress in achieving significant changes in this area, the interviewed stakeholders underlined that it is not only necessary to address masculinities, but also to do it in a way that accounts for the gendered construction men and boys, taking into account the plurality of their experiences. As the following interview extract shows when developing programs and interventions, addressing diversity in gendered experience is a crucial point to consider.

*I think the important thing is to get to the root of where it comes from and from there you can clarify a lot. But, if we consider that patriarchy has managed to build differentiated and unequal roles for men and women and that one way to maintain the status quo of the issue is precisely violence. That it can be more or less subtle, more or less obvious, but it is a way of maintaining power relations. But also, which men and which women? Because not all men are the same, also within the same category of “men” we cannot generalise because there are many other axes of oppression-privilege.* (Interview 14, man, expert in GBV, representative at a non-governmental association).

However, the advocacy to target gender inequality and restrictive gender norms also generated different perceptions among the professionals: although they observed the need to work on masculinities from a gender relational perspective, at the same time we identified a certain reluctance to this work justified by a lack of resources. Some professionals, especially those working with young people who have suffered from GBV, showed a clear concern: if working with boys and men on masculinities is funded by the same gender equality policies, this work may result in losing resources for organisations working with women survivors. Another critical position about working on masculinities is related to the workload involved, its responsibility and tasks. Some professionals stated that the social pedagogy of educating men about GBV and gender-related issues should mainly fall under men’s spaces and responsibilities, as seen in the following excerpt from an interview.

*What happens is that, of course, we can work on the new masculinities, but we believe that there has to be spaces for men where they themselves work on it. The pedagogical responsibility of educating masculinities does not have to fall on women, so to speak.* (Interview 8, woman, youth educator and GBV prevention worker).

Working through group workshops was deemed essential to create opportunities for reflection among men. Several professionals emphasized the significance of starting by challenging the beliefs, myths, and taboos associated with the societal construction of dominant masculinity. From there, it was crucial to directly address the mechanisms that reinforce privileges and power dynamics, including those that are subtle and go unnoticed in everyday life, commonly referred to as “micromachismos” within the scope of this study.

*I mentioned this on one occasion, not as a speaker, but in a round table discussion [in a colloquium after a conference]. And there were several high school teachers from the area who came to tell me: “Hey, this is interesting”. Because of course, they also wanted to work on these issues with the kids in their high schools. And this type of strategy is a hook. In the end, there’s no secret to it. It’s simple, let’s sit here and talk about how we flirt because many times as men we only talk about cars, soccer, and little else… Well, we are really going to question here how we are doing this kind of thing* (Interview 21, man, expert on masculinities).

*We also work on relationships between boys and girls. And we have to work on gender roles and stereotypes. Also giving them the weight that this has on them [men]. Because they are also oppressed; they can’t get out [easily] of the hegemonic masculinity, because whoever gets out is punished by the rest. But they also have to be aware of the privilege* (Interview 23, woman, representative in a feminist organization).

#### Connecting GBV prevention to gender-transformative approaches

When discussing the strategies used to construct a GBV prevention discourse, the professionals pointed out that better results are achieved when the educational methods and tools developed to understand the gendered influence are directly connected to real situations experienced by young people and when dynamics are implemented that facilitate greater involvement of young people in their own design, as this also enhances the possibility of training them as agents of change. However, the effectiveness of this approach is restricted when interventions are limited to sporadic actions, which do not facilitate a systematic and structural approach to violence prevention.

*We focus more on what they experience. Because in the end, I believe that it is the only way you can do it and teach them that there really is inequality (…) But, at those ages, they will not reflect on that topic with a two-hour talk.* (Interview 14, man, GBV expert and youth educator, representative at a non-governmental association).

Furthermore, the professionals affirmed that prevention work is important to stimulate a sense of responsibility and awareness while considering the reaction of young people, especially boys. However, effective GBV prevention requires more strategic educational work on masculinities. This was emphasised by some professionals as a central point to guide actions towards a transformative change of social norms in the study context. At a practical intervention level, when gender-sensitive approaches are not directly incorporated into existing curricula, the GBV prevention discourse could even contribute to exacerbating reactions of distance and disengagement in boys and men. In any case, concerning this issue, the professionals focused on the need to avoid restrictive approaches that might generate resistance against GBV prevention. While the findings indicate that some professionals believe that all boys and men have the potential to be aggressors due to the patriarchal nature of society, most of them emphasized the importance of avoiding defensiveness among men and boys in order to achieve more effective results with programs and interventions. They recognized that singling them out and attacking them would not be productive. Instead, the results suggest that taking a positive approach is more effective when addressing these issues.

*We try to explain to them that society as a whole is sexist. It is not that you are sexist. You are not to blame. You are here and you assume it. You need to break it down if you want to change it. That is why we do not focus on that. You do not have a problem, we all have a problem.(…) They [boys and men] are very grateful to see a different point of view than what they normally see. Because, of course, in the end, you’re being confronted with situations where you’re guilty; and then someone comes along and says no. You decide what to be. You decide what to be. You decide what to be. You decide what to be. You decide what to do (Interview 8, woman, GBV prevention worker).*

*Here* [*at the* foundation] *we do a quarterly activity with boys and girls, one every quarter. Then what we do throughout the school year is to go to the high schools. Mostly they are workshops that promote good treatment relationships and prevent gender violence, but we always talk in a positive way, good treatment relationships, egalitarian relationships* (Interview 3, woman, representative in a feminist organization).

In the same sense as engaging men and boys in GBV prevention, some stakeholders agreed on showing the advantages of egalitarian relations and supporting anti-violence masculinities, as well as stressing the limitations of adhering to normative sexist standards.

*Giving them examples, showing them results on the advantages of having a more equal and respectful society with women or with people with certain sexual identities that are different to the binomial ones. It needs to go through education (…) We need to start to change the discourse to value those other ways of being a man, that are careful, caring, treat people well, equal relationships, loving, sensitive. But also empower men so they can be who they want to be. So they do not have to show how macho they are all the time. Because that demonstration of masculinity is made towards a group of peers, right? It is the peers, the other men, who really validate that behaviour, and the masculinity of each person.* (Interview 3, man, gender equality and masculinities worker).

The professionals also underlined the importance of developing bystander interventions on how to engage men and boys in recognizing and preventing GBV actively. However, the challenge for the professionals in stimulating bystander intervention lies in having to deal with men as peers: addressing GBV behaviours, questioning group pressure and suggesting different values and attitudes from the mainstream and what is socially expected as boys “being men”. This act of “leaving their comfort zone” constitutes, according to the interviewed professionals, not only a challenge for the interventions but also a responsibility, since it finally entails letting go of many men’s privileges.

*Statistically, it is impossible that it will never happen: “No, I have never had a friend who has not said a sexist comment”. That is where we have to [act]. That is where you have to really show if you are not sexist, or if you are and want to change it. Are we all born sexist? No, but we have been educated to be sexist.* (Interview 14, man, GBV expert, representative at a non-governmental association).

*Men have a responsibility to break down all that sexist discourse, and we have to dare to confront the men in our circle who have this type of behaviour. Men also have the responsibility to review what our behaviours are, and to empathise and worry about knowing what the reality of women is. That means leaving, to a large extent, the comfort zone, and well, giving up a lot of privileges.* (Interview 22, man, gender equality and masculinities worker).

## Discussion

This study was designed to provide insights by professionals of different governmental and non-governmental organisations related to GBV prevention in young populations. Our findings show that professionals involved in GBV prevention perceive a contradiction between young people’s GBV awareness and their (lack of) identifying control practices as GBV in their interpersonal relationships. Both young women and men tend to identify GBV primarily as physical abuse by partners and sexual abuse by strangers. Due to their privileged position as men, the professionals emphasized that it is harder for boys to self-criticise their role in the perpetuation of sexism and GBV behaviours. In order to prevent GBV, the results point to the need to apply gender-transformative approaches, especially on how masculinities are socially constructed. To do this, the professionals consulted underline the relevance of using participatory approaches and educational strategies that connect directly with real situations experienced by young people, in order to deal with daily sexist attitudes and behaviours which are normalised and not identified as harmful practices. Barriers and enablers are identified not only at an interpersonal and organisational level, but also at a societal level.

The approach to the experiences of the interviewed professionals has also allowed us to gain first-hand knowledge of what they consider to be the main challenges in addressing GBV at an individual and interpersonal level in the study context, starting by detecting how different forms of violence operate in young populations. Consistent with the scientific literature, our findings align with the view held by professionals that young people often have a limited understanding of violence, initially perceiving it solely as physical acts and abuse [37]. This implies that programmes and interventions must go beyond this lay knowledge of violence understood as physical assault, and approach a range of other attitudes and practices, including gendered controlling or threatening behaviours [38].

Regarding the new forms of violence, our findings clearly point to the need to pay attention to the control and surveillance through digital communication technologies. We are currently facing “cyber violence”, an emerging field of action, which highlights the need to develop more holistic programmes [39–41]. In this sense, our results are in line with the study by Messing et al. [42], which shows that one of the main difficulties in addressing this type of violence lies in the subjective nature of online interactions, as highlighted by the fact that young women exposed to cyber-violence themselves do not have a clear idea of the boundaries of stalking or harassment or the implications of other technology-based abusive behaviours, such as the distribution of sexually compromising images or data (real or fake) on the Internet. Although this notion may reinforce gender role stereotypes associated with gender-based violence, it also underscores the importance of placing emphasis on recognizing and addressing the involvement of young individuals in cyber-violence. Thus, it is crucial to ensure they comprehend the repercussions of their actions.

Although our results suggest that the programs and interventions that are being implemented are not as effective as expected, perhaps not so much due to lack of resources, but because on many occasions they do not address the key problems of the moment and are adapted to the experiences to which young people, also reveal other recommendations at the organizational level. In the study context, the fact that many of these educational interventions are not included in the school curriculum, and are developed as sporadic initiatives, does not facilitate to examine of the essential elements of the structure, content, and implementation of an intervention, which provides us with knowledge about its mechanisms of action. In this sense, research evidence in this field shows that long-term initiatives are more likely to achieve lasting changes than short-term initiatives [11]. On the other hand, we also observed that another limitation at the organizational level has to do with the fact that the contents demanded by educational institutions focus on victims and aggressors from an individual perspective, instead of being able to propose multicomponent approaches based on community-based interventions. However, in order to develop these more systemic approaches, it is necessary to move towards preventive and health promotion actions from a more collaborative perspective at an interdisciplinary and intersectoral level [43–45].

Our findings also show that stakeholders suggest designing educational programmes on the deconstruction of gender expectations and the harmful behaviours underpinning GBV, but going beyond individual components (i.e., awareness of rights, understanding personal practices) with partners and peers. In this sense, literature has shown that the approach to these contents in preventive strategies should focus on community and structural social determinants, such as school programmes, social marketing and media, instead of focusing solely on individual perpetrators and victims [46]. Research has consistently shown that implementing interventions simultaneously at the different levels of influence acting on people is more effective [3]. Thus, although our findings pivot especially on the individual and interpersonal levels, as we expected given the specific object of the study, direct or indirect connections also emerge from the results at the community, institutional and sociocultural levels in which people live and develop. This underlines the need to address the development of actions in a coordinated and collaborative manner with different actors, both governmental and nongovernmental, while still considering this type of violence as a public health problem [47, 48].

Another issue that our results highlight is the importance of developing practical pathways to address gender norms with a further engagement of boys in taking an active role against GBV, that is, not only as allies but as agents of change in gender equality efforts [12, 49, 50]. This point involves the need to change the approach, encouraging gender-transformative programmes to be developed to address GBV [5]. In this regard, the programmes and interventions that show the best results are those that address the relationship between violence and hegemonic masculinities as critical factors intertwined in the process of achieving greater levels of gender equality [24]. In addition, recognizing the important role that men and boys play in progressing towards gender equality is also contributing to defining this critical issue in a broader sense, maintaining the emphasis on power relations, not only men over women, but also of men and boys over other men [24]. This requires a deeper understanding of the impact of masculinities on different forms of violence such as those against LGBTQ identities, as well as addressing their intersection with other social identities such as class and ethnicity.

The literature shows that boys and young men have specific health needs that can be improved through school and environmental health programs developed with a gender approach and focusing on social determinants [51]. However, our findings echo that involving boys to a greater extent has generated a growing debate about whether it is more effective to work only with boys, i.e., in gender-specific programmes. Although there is some evidence in this line of research, the results should be taken with caution, not only because of the limited number of studies assessed with this approach, but also because of the variability of the results [52]. In fact, of the 18 studies identified by Steward et al., [53]. focused on boys and men, only four of them reported significant results. Furthermore, only two revealed changes in norms and/or stereotypes related to men, but not in relation to personal norms, and important discrepancies were also found in relation to attitudes towards women.

When working at an individual and interpersonal level in education programmes and fostering engagement with men and boys, scientific literature highlights that interventions have a greater chance of positive results when using one or more additional strategies such as peer-based learning or involving participants in the design process [54, 55]. In this sense, our results also draw attention to the importance of the content being closely linked to daily situations lived by young people, in which they feel reflected, as this enhances the meaningful learning experience.

### Limitations

Our results are specific to the Spanish context, particularly the city of Madrid and its metropolitan area. However, we aimed to include participants with experience working in various territories within this context to gain insights into their perspectives on a culturally diverse young population. While we did not directly consider other social variables such as sexual orientation or gender identity, we selected participants in a way that the groups or organizations they represented would potentially provide a broader view from an intersectional gender perspective. This approach allowed us to capture the diverse work carried out by these groups in relation to the topic under study.

It is important to note that our findings are based solely on the professionals’ discourses, and therefore, future research could benefit from analyzing their practices and comparing their views on preventive action with those of young program users. By incorporating the perspectives of young users, a more comprehensive understanding can be gained, shedding light on the effectiveness and impact of these programs from the viewpoint of the individuals they aim to intervene.

## Conclusions

The results show that among the main challenges identified by stakeholders in relation to preventive strategies against gender-based violence in young populations, there is a need to focus on transformative programmes within educational settings. The findings show that the specific programs and interventions being implemented may not be as effective as expected, not so much because of a lack of resources, but mainly because they do not address the key issues needed at this time. The results underline that these programmes should emphasise equitable gender relations, work on questioning gender norms and incorporate content on anti-violence masculinities. Importantly, the general engagement of young people can be more feasible when strategies, such as peer learning or involving them in the design process itself, are established. In particular, this aspect is seen by professionals consulted as a mechanism to facilitate interventions that are more connected to young people’s everyday situations. Precisely in relation to the need to bring programme contents closer to young people’s experiences, the results draw attention to the need to analyse the impact of new forms of violence in greater depth, especially those that occur through information and communication technologies. Among other implications for policy and practice, the results point to the need to articulate interventions designed to work simultaneously at different levels of action.

## Electronic supplementary material

Below is the link to the electronic supplementary material.

Additional file 1- coding tree

## Data Availability

The datasets generated and/or analyzed during the current study are not publicly available due to the personal information and sensitive information from stakeholders, comprehended in the interviews and which could in theory be might be traced back to individual respondents. The data is only available on site after contact with the corresponding author on reasonable request, to ensure data access complies with the procedures of the General Data Protection Regulation (GDPR).

## Abbreviations

GBV: gender-based violence
VAW: violence against women
LGBTQ: lesbian, gay, bisexual, transgender and queer
