# Supplementary material for: Listening to stakeholders in the prevention of gender-based violence among young people in Spain: a qualitative study from the positivMasc project
Source: BMC Womens Health. 2023 Jul 26;23:393. doi: 10.1186/s12905-023-02545-3 (PMC10373224; doi:10.1186/s12905-023-02545-3)
Supplement: Supplementary file 1 — Additional file 1- coding tree [file 12905_2023_2545_MOESM1_ESM.docx]

**POSITIV MASC CODE TREE**

**Stakeholders’ interviews**

| *VAW: Violence Against Women | *GV: Gender Violence |
| --- | --- |

| **CATEGORY** | **CODE** | **DESCRIPTION** |
| --- | --- | --- |
| **GENDER DISCOURSES** | **Masc Social discourses** | General perceptions of being male, masculine. Those of the general population, our parents, our friends, the media, etc. |
|  | **Fem Social discourses** | General perceptions of being female, feminine. Those of the general population, our parents, our friends, the media, etc. |
|  | **Masc Organization discourses** | How does the organization understand masculinity. What is the organization’s perspective on managing masculinities. |
|  | **Fem Organization discourses** | How does the organization understand femininity. What is the organization’s perspective on managing femininities. |
|  | **Masc Subjective discourses** | Subjective perceptions and discourses of the person interviewed of the organization regarding of being male, masculine. |
|  | **Fem Subjective discourses** | Subjective perceptions and discourses of the person interviewed of the organization regarding of being female, feminine. |
|  | **Gender Organization discourses** | Discourses and perceptions of the organization regarding of gender, in general. What is the organization’s perspective around the concept of gender. |
|  | **Gender Social discourses** | Social discourses and perceptions regarding of gender, in general. |
|  | **Gender Subjective discourses** | Subjective perceptions and discourses of the person interviewed regarding of gender, in general. |
|  | **Gender and sexuality Social discourses** | Discourses around gender and sexuality. Ex: sexual stereotypes depending gender, gendered sexual practices outside and inside an intimate relationship, etc. |
| **GV AND VAW DISCOURSES** | **GV Organization discourses** | How does the organization understand GV. What is the organization’s perspective on managing GV. |
|  | **GV Social discourses** | Comments about the social perception of GV regarding the organization’s work and experience. |
|  | **GV Subjective discourses** | Subjective perceptions and discourses of the person interviewed regarding of gender violence. |
|  | **VAW Organization discourses** | How does the organization understand VAW. What is the organization’s perspective on managing VAW. |
|  | **VAW Social discourses** | Comments about the social perception of VAW regarding the organization’s work and experience. |
|  | **VAW Subjective discourses** | Subjective perceptions and discourses of the person interviewed regarding of VAW. |
|  | **Feminism and VAW** | Opinions about feminism and its role in VAW. |
|  | **GV-VAW perpetrators** | Who are the responsible of GV-VAW. It focuses on the “who take part” and could be at different levels. They can be guilty or instigators. Ex: The men, the social media, the educational institutions, etc. |
| **STRATEGIES TO PREVENT GV and VAW** | **Strategies in place GV/VAW** | Description of the strategies and actions implemented by the organization to prevent GV-VAW. |
|  | **Assessment of strategies**  **GV/VAW** | Achievements and failures as identified in the implementation of strategies to prevent VAW and GV. |
|  | **Proposals to prevent VAW/VG** | Other proposals that could be implemented to prevent VAW. |
|  | **Engagement and participation of men** **GV/VAW** | Opinions about how men could or should be part of the prevention of VAW. How to involve men and which role could they play. |
|  | **Engagement and participation of women** **GV/VAW** | Opinions about how women could or should be part of the prevention of VAW. How to involve women and which role could they play. |
|  | **Institutional difficulties/obstacles prevention GV/VAW** | Comments about the limits of public institutions to prevent GV-VAW. Ex: lack of funding, legal gaps, influence of the political context, etc. |
|  | **Social difficulties/obstacles** **GV/VAW** | Comments about the social ideas or practices that stop the progress against VAW. |
| **STRATEGIES TO PROMOTE POSITIVE MASCULINITIES** | **Strategies in place Masc** | Description of the strategies implemented by the organization to promote positive masculinities. |
|  | **Assessment of strategies Masc** | Achievements and failures as identified in the implementation of strategies to promote positive masculinities. |
|  | **Proposals to promote Masculinities against VAW** | How can be promoted these masculinities against VAW? Other proposals that could be implemented or are being implemented by other organizations to promote masculinities against VAW. |
|  | **Institutional difficulties/obstacle Masc against VAW** | Comments about the limits of public institutions to promote masculinities against VAW. Ex: lack of funding, legal gaps, influence of the political context, etc. |
|  | **Social difficulties/obstacles Masc against VAW** | Comments about the social ideas or practices that stop the progress towards positive masculinities. |
